# Pilot Study on the Efficiency of the Biostimulation with Autologous Plasma Rich in Platelet Growth Factors in Otorhinolaryngology: Otologic Surgery (Tympanoplasty Type I)

**DOI:** 10.5402/2011/451020

**Published:** 2011-06-20

**Authors:** María Luisa Navarrete Álvaro, N. Ortiz, L. Rodriguez, R. Boemo, J. F. Fuentes, A. Mateo, P. Ortiz

**Affiliations:** ^1^Department of Otorhinolaryngology, Vall d'Hebron Hospital, Autonomous University of Barcelona, 08035 Barcelona, Spain; ^2^Blood-Tissues Bank, Vall d'Hebron Hospital, Autonomous University of Barcelona, 08035 Barcelona, Spain

## Abstract

When otologic procedures that involve tympanic membrane repairs are performed, biomaterials or biological tissues as normal as grafts are used. At the moment, biological material from the own patient is used with varying success rates. The procedure used and the patient's tissue repair capabilities tend to determine the outcome. We present a preliminary study on tympanic membrane perforation repairs using an autograft obtained by manipulating platelet degranulation and the coagulation cascade and reinforced with a seal using platelet growth factors. We present three cases in which we used this procedure. The results will be valued based on the tympanic perforation closure index. With this study, we want to assess the effectiveness of tympanic perforation repairs with this technically simple method. If this method was objectively proved to be effective, it would lead to lower patient morbidity and sanitary costs.

## 1. Introduction

Replacement therapy using tissue biostimulation involves a series of procedures that try to re-establish tissue metabolism and function. Biostimulation is the biological activation of the fibroblast's anabolic functions. Proline, lysine, and glucosamine, after interacting with growth factors, evolve into collagen, elastine, and hyaluronic acid. 

Fibroblast activation occurs by influence of growth factors or GF. These are small biologically active proteic fragments that belong to the cytokine group [1]. 

Growth factors can be produced and stored by multiple tissues and cell classes, among them platelets. Platelet-rich plasma (PRP) is obtained by blood extraction and manipulation, with the end goal of obtaining platelet degranulation and protein release. Among these proteins are those that contain growth factors (PRGFs). 

Otologic surgical procedures that attempt to repair tympanic membrane defects currently use a graft of biological material from the own patient. Tragal perichondrium obtained from the ipsilateral ear is typically used, with mixed results. Tympanic membrane graft migration explains some of these in situ failures, in conjunction with natural events that take place in the membrane. 

It can be inferred that the using substances that promote quick and effective growth could avoid graft migration by their direct cohesion with tympanic remains. In the past years, there have been several attempts to use tissue adhesives in Otolaryngology [2]. The use of fibrinogen derivates would allow us more options for Otologic and Otoneurologic surgery, among other ENT areas.

## 2. Materials and Methods

Platelet rich plasma, or PRP, is obtained by extracting 10 cc of peripheral blood, which is then centrifuged at room temperature to produce platelet degranulation. The thrombin fractions are then isolated so thrombin can catalyze the conversion of fibrinogen to fibrin and promote the start of the coagulation cascade, platelet degranulation, and PRGF growth factors release. That way a platelet and fibrin STBA clot for treatment and plasma rich in GF proteins and thrombin. 

We present the preliminary results of three cases in a prospective study of patients referred to our center with inactive central tympanic membrane perforation. 

The procedure is explained thoroughly to all the possible participants in the study, both in the Otorhinolaryngology outpatient clinic and in the Blood and Tissues Bank. Every procedure is authorized by a signed informed consent form. 

**Figure 1:**
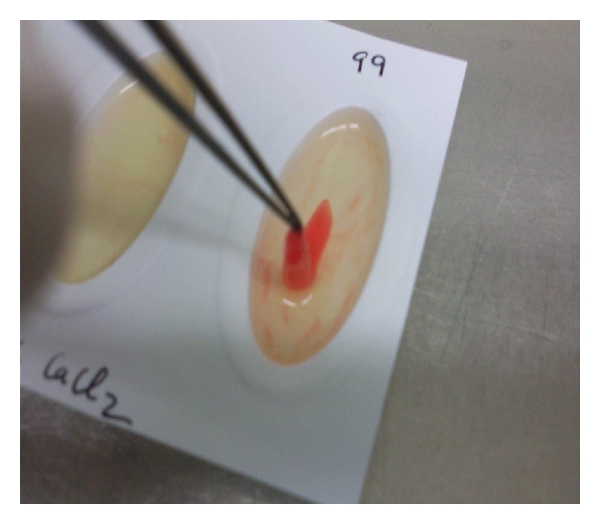
Thrombus-gel resulting from the PRP obtaining procedure.

Patients with known autoimmune, active neoplastic, atopic or active otic conditions, and those currently under immunosuppressive treatments were excluded. The surgical procedure is performed with an imbibition of the PRP around the perforation. An uplay seal of the tympanic perforation is then performed to guarantee the tissue growth progression with a thrombus-gel guide (Figure 1). The results are valued by the level of tympanic closure index.

## 3. Results

**Figure 2:**
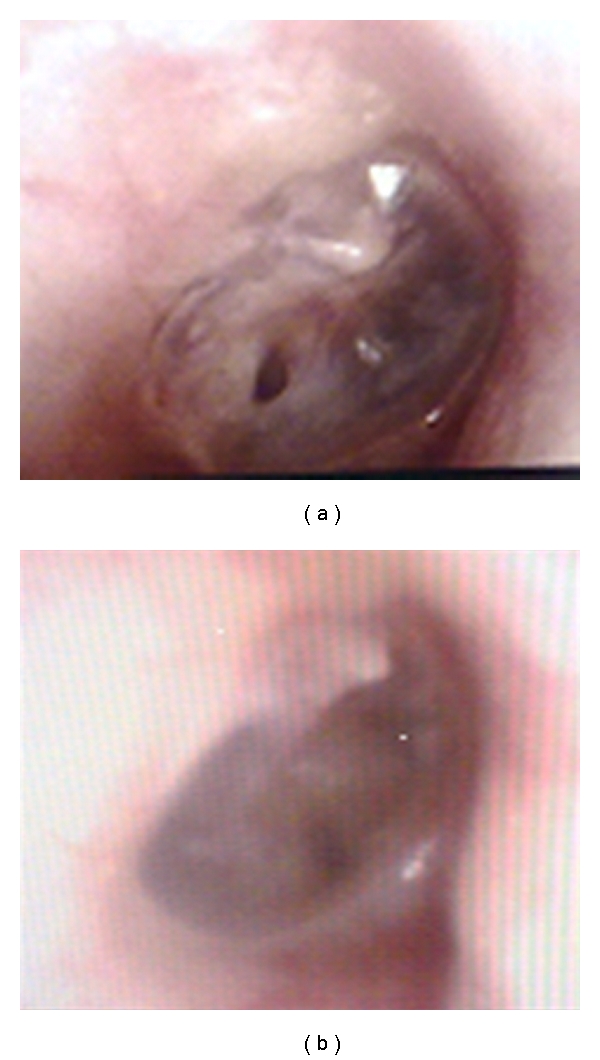
Pre- and postoperational otoendoscopic images of case 1.

**Figure 3:**
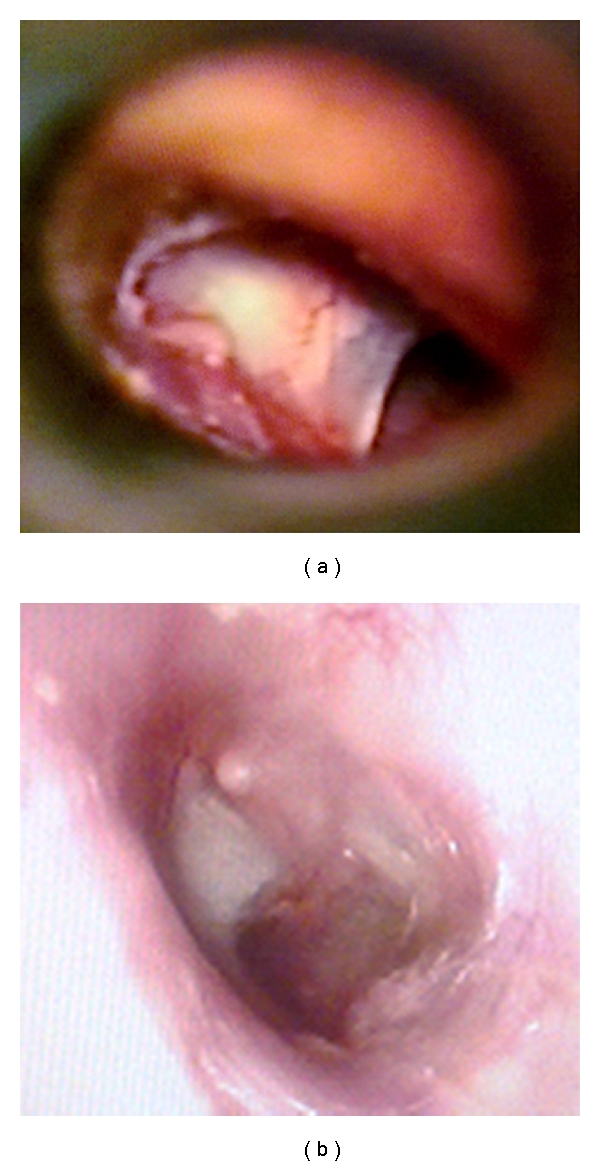
Pre- and postoperational otoendoscopic images of case 2.

**Figure 4:**
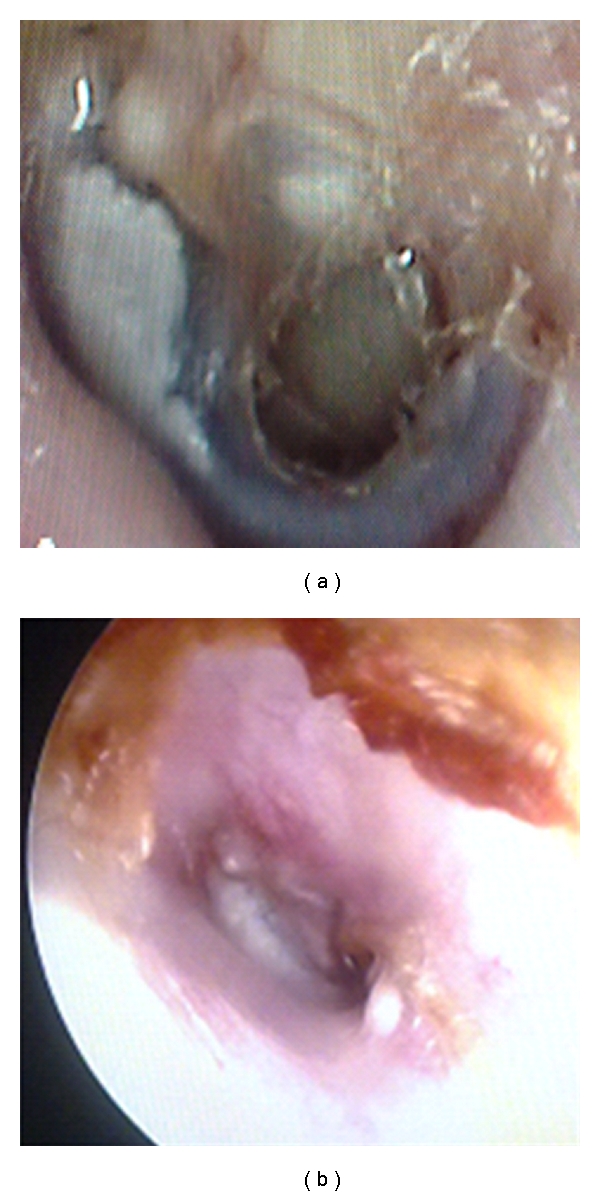
Pre- and postoperational otoendoscopic images of case 3.

In all three cases, the perforation closed in its entirety (Figures 2, 3, and 4).

## 4. Discussion

Biostimulation is the biological activation of the fibroblast's anabolic functions. Fibroblast activation occurs by influence of growth factors or GF. These are small biologically active proteic fragments that belong to the cytokine group. Cytokines join membrane receptors to activate or inhibit celular functions. A type of cell regeneration, specific to the tissue where the cells are located, is produced. The TGF transformations factors either increase cell growth, in the case of TGF-alfa, or decrease it in the case of TGF-beta.

The PDGF, TGF, VEGF, IGF, FGF, and HGF growth factors can be produced and stored by multiple cells and tissues, among them platelets. They intervene in intercellular communication, informing the cells of their location at any given moment, the type of cells that surround them, and their needed function at that moment [1]. The requirements of a RPR system (Platelets Growth Products) are: small volumes of blood, increasing the base platelet levels 3-4 times, platelet viability, and the relation between platelet count and GF proteins. This way a platelet and STBA fibrin clot is obtained to be used as treatment in injured zones, as well as GF proteins and thrombin rich plasma [3, 4]. The PDGF growth factor stimulates cell proliferation, fibroblast chemotaxis, and collagen synthesis. The growth-transformation factor, TGF, controls cell proliferation, has intrinsic inflammatory ability, and induces extracelular matrix production and tissue repair. The epidermic growth factor or EGF promotes keratinocyte production, stimulates angiogenesis, fibroblast chemotaxis, and a provisional matrix formation. In short, GF produces: regeneration, in new functional tissue that is identical to the original, and repair in a scar [5, 6]. 

When otologic procedures that involve tympanic membrane repairs are performed, biomaterials or biological tissues as normally used as grafts. There is a constant search for materials that meet certain requirements: efficacy, low cost, safety, and a structural composition that is as similar as human tissue as possible. Currently, the patient's own biological material obtained from the ipsilateral tragal perichondrium or temporal fascia is used as graft. The difference in results is mostly due to the incision closure technique and the patient's own tissue repair response.

The migration mechanisms intrinsic to tympanic membrane grafts when they interact with the membrane's own natural mechanisms explain some failures at in situ placement. Migration could be due to mitotic activity at the center of a structure with a central to periphery growth pattern, that is, centripetal to the cell generation center. It could be inferred that the use substances that promote fast and effective growth could avoid graft migration by their direct cohesion with tympanic remains. In the past years, there have been several attempts to use tissue adhesives in Otolaryngology. In our line of research, we attempt to treat tympanic perforations with an autograft, obtained by manipulating platelet degranulation and the coagulation cascade and reinforced with a seal using platelet growth factors. These platelet growth factors play a fundamental role in the situation. Platelets transport these factors and release them where there is tissue damage. Platelets also transport proteins essential to tissue repair and regeneration, some that originate in their cell precursors (megakaryocyte) and others that are captured from the surrounding plasma by endocitosis. The most important thing to keep in mind during this study is that platelets are fairly easy to obtain [7]. 

The large number of patients that we tend to in our university hospital become a stimulus to search for new surgical alternatives that bring about better results for the patient, while at the same time lowering the number of failures. By achieving more affordable techniques, we ultimately lower patient care costs and surgical time overbooking.

We're currently in the middle of a prospective, longitudinal, and observational study of patients referred to the ENT outpatient clinics of our hospital for tympanic perforation, independent to the cause of the condition (posttraumatic, chronic otitis media, past surgical failures). After verifying that all the possible participants in the study meet the inclusion criteria, the procedure and the followup needed afterwards is explained thoroughly to them, both in the Otolaryngology outpatient clinic and in the Blood and Tissue Bank. The results will be valued based on the tympanic perforation closure index, which has been satisfactory in our preliminary experience.

In summary, we present a minimally invasive Type I Tympanoplasty technique performed using a PRP graft from the own patient, obtained at the Blood and Tissue Bank. We value tympanic perforation repairs positively, using a technically simpler uplay method with lower morbidity for the patient and a perforation seal guarantee. When the procedure's efficacy is proved in a larger sample, it would entail a lower intrahospital and outpatient cost of tympanic perforation repair.
